# P2Y_2_ Receptor Promotes High-Fat Diet-Induced Obesity

**DOI:** 10.3389/fendo.2020.00341

**Published:** 2020-06-03

**Authors:** Yue Zhang, Carolyn M. Ecelbarger, Lisa A. Lesniewski, Christa E. Müller, Bellamkonda K. Kishore

**Affiliations:** ^1^Department of Veterans Affairs Salt Lake City Health Care System, Nephrology Research, Salt Lake City, UT, United States; ^2^Departments of Internal Medicine, University of Utah Health, Salt Lake City, UT, United States; ^3^Jiangsu Key Laboratory of Pediatrics, Nanjing Medical University, Nanjing, China; ^4^Division of Endocrinology and Metabolism, Department of Medicine, Center for the Study of Sex Differences in Health, Aging, and Disease, Georgetown University, Washington, DC, United States; ^5^Department of Veterans Affairs Salt Lake City Health Care System, Geriatric Research, Education and Clinical Center, Salt Lake City, UT, United States; ^6^Department of Nutrition and Integrative Physiology, University of Utah Health, Salt Lake City, UT, United States; ^7^Center on Aging, University of Utah Health, Salt Lake City, UT, United States; ^8^PharmaCenter Bonn, Pharmaceutical Institute, Pharmaceutical and Medicinal Chemistry, University of Bonn, Bonn, Germany

**Keywords:** purinergic signaling, obesity, adipose tissue, AR-C 118925, insulin resistance, inflammation, glucose homeostasis, lipid tolerance

## Abstract

P2Y_2_, a G protein-coupled receptor (R), is expressed in all organs involved in the development of obesity and insulin resistance. To explore the role of it in diet-induced obesity, we fed male P2Y_2_-R whole body knockout (KO) and wild type (WT) mice (B6D2 genetic background) with regular diet (CNT; 10% calories as fat) or high-fat diet (HFD; 60% calories as fat) with free access to food and water for 16 weeks, and euthanized them. Adjusted for body weights (BW), KO mice consumed modestly, but significantly more HFD vs. WT mice, and excreted well-formed feces with no taint of fat or oil. Starting from the 2nd week, HFD-WT mice displayed significantly higher BW with terminal mean difference of 22% vs. HFD-KO mice. Terminal weights of white adipose tissue (WAT) were significantly lower in the HFD-KO vs. HFD-WT mice. The expression of P2Y_2_-R mRNA in WAT was increased by 2-fold in HFD-fed WT mice. Serum insulin, leptin and adiponectin levels were significantly elevated in the HFD-WT mice, but not in the HFD-KO mice. When induced *in vitro*, preadipocytes derived from KO mice fed regular diet did not differentiate and mature as robustly as those from the WT mice, as assessed by cellular expansion and accumulation of lipid droplets. Blockade of P2Y_2_-R by AR-C118925 in preadipocytes derived from WT mice prevented differentiation and maturation. Under basal conditions, KO mice had significantly higher serum triglycerides and showed slightly impaired lipid tolerance as compared to the WT mice. HFD-fed KO mice had significantly better glucose tolerance (GTT) as compared to HFD-fed WT mice. Whole body insulin sensitivity and mRNA expression of insulin receptor, IRS-1 and GLUT4 in WAT was significantly higher in HFD-fed KO mice vs. HFD-fed WT mice. On the contrary, the expression of pro-inflammatory molecules MCP-1, CCR2, CD68, and F4/80 were significantly higher in the WAT of HFD-fed WT vs. HFD-fed KO mice. These data suggest that P2Y_2_-R plays a significant role in the development of diet-induced obesity by promoting adipogenesis and inflammation, and altering the production of adipokines and lipids and their metabolism in adipose tissue, and thereby facilitates HFD-induced insulin resistance.

## Introduction

Overweight and obesity affect about 30% of the world population on an average, and is much higher in the United States (1). The reported prevalence of overweight and obesity among US Veterans is from 40 to 73% (2, 3). Surprisingly, the combined overweight and obesity in active duty US military personnel rose to more than 60% between 1995 and 2008 (4). Experimental, clinical and epidemiological data link obesity to the development of type 2 diabetes mellitus (T2DM), metabolic syndrome, chronic kidney disease, cardiovascular and cerebrovascular complications, and hypertension (5, 6). In addition, obesity is known to be associated with higher incidences of cancer (7). Because of these serious consequences, 1 in 5 deaths in the United States is associated with obesity. Furthermore, cost and socioeconomic burden of overweight and obesity to individuals and to the nations are huge (8–10). In view of these, in June 2013, the American Medical Association recognized obesity as a disease (11).

Diet-induced obesity or consumption of more calories than what one can utilize or burn (energy surplus) is the most common form of obesity. Although a preventable condition, in reality it is difficult to prevent or treat diet-induced obesity. The anti-obesity drugs fall into three main categories: (i) those that suppress appetite by acting on the brain; (ii) those that interfere with body's ability to absorb specific nutrients in the food, such as fat; and (iii) those that boost the body's metabolic rate. The first two categories of drugs have serious side effects, thus limiting their widespread use. In addition, the US Food and Drug Administration (FDA) withdrew some of them (e.g., sibutramine). Although drugs that boost the body's metabolic rate offer a better alternative, potentially efficacious and safer drugs are not currently available for the treatment of overweight and obesity. Although some of the newly introduced anti-obesity drugs are relatively safer, their long-term efficacy is not well-established, especially in terms of long-term usage (12, 13). Hence, there is an unmet need for determining novel drug targets for the development of efficacious therapies with non-lethal correctable side effects, for use in the clinics or in general population on a wider scale.

The study of purinergic signaling, which is mediated by extracellular nucleotides (ATP/ADP/UTP) through P2 receptors (P2Y and P2X series) is an emerging field with a vast potential for the development of novel therapies for a wide spectrum of disease conditions, in addition to unraveling physiological and pathophysiological mechanisms (14–16). For many years, our interest has been on the role of purinergic signaling in the kidney in health and disease. Specifically, we have been investigating the roles of ATP/UTP-activated P2Y_2_ and ADP-activated P2Y_12_ receptors in renal physiology and pathophysiology, with respect to water balance disorders, such as acquired nephrogenic diabetes insipidus or NDI [reviewed in Kishore et al. (17, 18)].

P2Y_2_ is a G protein-coupled receptor that selectively responds to ATP or UTP, but not to ADP. Activation of P2Y_2_ receptor results in phosphoinositide hydrolysis and release of intracellular free calcium, and diacylglycerol (DAG), and the ensuing activation of protein kinase C (PKC) among others molecular events (19, 20). Currently, there are no FDA-approved drugs for selectively blocking P2Y_2_ receptor. Hence, we have been using P2Y_2_ receptor knockout (KO) mice in our studies. In addition to its effect on renal water handling, we and other investigators have shown that P2Y_2_ receptor plays a role in renal sodium handling [reviewed in (21)]. During our efforts to understand how obesity affects renal handling of sodium in the P2Y_2_ KO mice, as early as in 2012, we serendipitously discovered that genetic deletion of P2Y_2_ receptor confers significant resistance for the high-fat diet (HFD)-induced obesity. Our studies revealed that this resistance is not due to less intake of food or lack of intestinal absorption of fat in the KO mice. On the other hand, cellular and molecular analyses revealed that the KO mice are able to handle energy metabolism in a different way that does not result in obesity. Specifically, we observed that P2Y_2_ receptor promotes obesity and insulin resistance by effects on adipogenesis and adipocyte metabolism. Here, we present experimental evidence for the role of P2Y_2_ receptor in adipogenesis and adipocyte metabolism and inflammation in white adipose tissue.

In view of the potential intellectual property issues related to our novel discovery made in 2012, and as mandated by the US Department of Veterans Affairs, in October 2012 we filed a provisional coversheet intellectual property protection to the United States Patent and Trademark Office (USPTO), which was followed by a regular patent application in 2013. After securing the priority date and protection for our discovery, we presented the key elements of our findings at the Kidney Week 2013 meeting (22), followed by two more meeting abstracts (23, 24). On May 1, 2014 our patent application was published online by the World Intellectual Property Organization, which is an open access information database. After establishing the originality by “prior art search” and significance of our discovery, in 2018, the USPTO granted us two patents comprehensively protecting our intellectual property (25, 26). Thus, our discovery on the potential role of P2Y_2_ receptor in promoting diet-induced obesity and insulin resistance pre-date the recent publications by other investigators related to this topic, which we discussed in the Discussion section.

## Materials and Methods

### Experimental Animals and Protocols

The animal procedures described here were approved by the Institutional Animal Care and Use Committee (IACUC) and Research Safety Committee of the Department of Veterans Affairs Salt Lake City Health Care System, and were conducted as per the guidelines of the US Public Health Service. Breeders of P2Y_2_ receptor knockout (KO) mice in the B6D2 genetic background and congenic wild type (WT) mice were originally obtained from Dr. Beverly Koller of the University of North Carolina at Chapel Hill, Chapel Hill, NC, United States (27, 28). These are whole body knockout mice generated by targeted mutagenesis of P2Y_2_ receptor in embryonic stem cells. Breeding colonies were established at the VA Salt Lake City Health Care System. KO and WT mice were bred and genotyped as described previously (29). Mice were subcutaneously implanted with microchip transponders for efficient identification and tracking (Locus Technologies, Mountain View, CA, United States). In the experiments described below, age-matched male WT and KO mice were used. Multiple animal studies were performed in generating the data presented here. Except during 24-h collections of urine and monitoring of food and water consumption, the mice were housed in groups in conventional cages with bedding. During metabolic studies, mice were housed in metabolic cages (1 mouse/cage) for a maximum of 48 h on each occasion. Irrespective of the housing conditions, unless prohibited by specific experimental manipulations, all mice had free access to food and drinking water. Mice were fed either regular rodent chow containing 10% calories as fat (control diet) or a high-fat diet (HFD) containing 60% calories as fat (HFD). The HFD was purchased from Research Diets, Inc., New Brunswick, NJ, United States (Catalog # D12492). It contains added lard and soybean oil as fat. At the end of the experimental period, mice were humanely euthanized and blood and tissues were collected for analysis as described below.

### Effect of Feeding High-Fat Diet in WT and P2Y2 KO Mice

Groups of age-matched male WT and P2Y_2_ KO mice were fed regular rodent chow (control diet; CNT group) or high-fat diet (HFD group) for up to 16 weeks. Body weights, 24-h water and food intake and urine output were determined once in every 2 weeks by placing the mice in metabolic cages. Feces samples were also collected periodically. Glucose tolerance and insulin tolerance tests were performed during the last 2 weeks of the experimental period on different groups of mice. Mice were euthanized humanely. Blood samples were collected from the inferior vena cava during necropsy. Serum was separated after blood clotting, centrifuged and clear supernatant was used for assays. Total body white fat and organs, such as liver and kidney were collected, weighed and either processed or frozen.

### Morphological Examination of Tissues

Freshly harvested tissues were fixed in 10% buffered formalin solution for a few days, then dehydrated and embedded in paraffin. Paraffin-embedded tissues were cut at a thickness of 5 μm, deparaffinized, rehydrated and stained with hematoxylin and eosin. Stained sections were examined under a Reichert light microscope, and digital pictures were taken with a Nikon 995 Coolpix camera.

### Assays on Serum or Plasma

Clear supernatants of serum were used for the determination of the following, using commercial assay kits and as per the manufacturers' instructions. Serum insulin and leptin were assayed using ELISA kits from Crystal Chem, Inc., Downers Grove, IL, United States. Serum adiponectin was assayed using EIA kit from the Cayman Chemical Company, Ann Arbor, MI, United States. Serum or plasma triglycerides and glycerol were assayed colorimetrically using kits from the Cayman Chemical Company. Serum free fatty acids were assayed using a kit from BioAssay Systems, Hayward, CA, United States.

### Glucose Tolerance Test

We followed a published and standard protocol to conduct glucose tolerance test [GTT; (30)]. Briefly, after fasting for 12 h with free access to drinking water, mice received an intraperitoneal injection of sterile glucose solution (1.5 g/kg body wt.) in 0.5 ml of USP grade water. During the GTT procedure, the mice were kept in warm environment to ensure adequate peripheral blood flow. Droplets of blood were obtained in conscious mice by gently pricking the dorsal pedal vein with a sterile needle. Using these droplets of blood, glucose levels were measured on a Contour Glucometer (Bayer HealthCare, LLC, Mishawaka, IN, United States). Blood samples were collected from each mouse prior to (0 min), and 30, 60, 90, and 120 min after intraperitoneal injection of glucose solution.

### Insulin Tolerance Test

We followed a published and standard protocol to conduct an insulin tolerance test [ITT; (31, 32)]. Briefly, immediately before conducting the ITT, the mice were fasted for 4 h with free access to drinking water. During the test period, mice were kept in a warm environment to ensure adequate peripheral blood flow. Baseline (0 min) blood samples were collected from the dorsal pedal vein by gently pricking the conscious mice with a sterile needle. Using the droplets of blood, glucose levels were measured on a Contour Glucometer. After that mice were given intraperitoneal injections of human insulin (Humalin® R-100, Eli Lilly, Indianapolis, IN, United States) at a dose of 1 U/kg body weight using an insulin syringe. Blood samples were collected at 20, 40, 60, 80, 100, and 120 min after insulin injection for glucose determination, using the same procedure described for the 0 min sampling.

### Determination of Relative mRNA Expression

Real-time RT-PCR approach was used to determine the relative mRNA expression of molecules of interest as described previously (33–35). These molecules are: P2Y_2_ receptor, insulin receptor, insulin receptor substrates (IRS-1 and IRS-2), GLUT-4 (glucose transporter type-4), TNFα (tumor necrosis factor-alpha), MCP-1 (monocyte chemoattractant protein-1), CCR2 (C-C chemokine receptor type-2), CD68 (cluster of differentiation 68), F4/80 (EGF-like module-containing mucin-like hormone receptor-like 1), LPL (lipoprotein lipase), HSL (hormone-sensitive lipase), ATGL (adipose triglyceride lipase), and CD36 (fatty acid translocase). Total RNA was extracted from the white adipose tissue by Triazol method, and traces of genomic DNA were removed and processed for RT-PCR by the methods established in our laboratory. After RNA was precipated, RNeasy Mini Kit and RNase-free DNase Set (Qiagen, Germantown, MD, USA) were used to remove traces of genomic DNA. cDNA was synthesized by SuperScript reverse transcription (Invitrogen, Carlsbad, CA, United States). Using gene specific primers (Table 1), real-time PCR amplifications were carried out on cDNA samples in Applied Biosystems 7,500 Real-Time PCR System (Foster City, CA, United States), with AmpliTaq Gold and SYBR Green for detection. Target gene expression was normalized to that of expression of the housekeeping gene β-actin. Data were expressed as target gene expression relative to the expression of β-actin. The relative gene expression level was normalized to the expression of β-actin and calculated using the comparative cycle threshold (ΔΔCt) method, as described by Yuan et al. (41).

**Table 1:** Nucleotide sequences of primer pairs used in real-time PCR.

**Gene**	**Accession no**.	**Primer position**	**Primer sequence**	**Amplicon Size, bp**	**References**
P2Y_2_ receptor	NM_001302347	618–638 756–773	GCGTTTCCTCTTCTACACCAA ACCAGCACCCACACAACC	155	Designed
LPL	NM_008509	583–604757–777	AAGCTGGTGGGAAATGATGTGG TTCTGCATACTCAAAGTTAGG	194	(36)
CD36	NM_001159558	794–814 707–726	GATGACGTGGCAAAGAACAG TCCTCGGGGTCCTGAGTTAT	107	(37)
HSL	NM_010719	1083–1103 1132–1150	GGAGCACTACAAACGCAACGA TCGGCCACCGGTAAAGAG	67	(38)
ATGL	NM_001163689	724–743 849–868	AACACCAGCATCCAGTTCAA GGTTCAGTAGGCCATTCCTC	144	(39)
Insulin receptor	NM_001330056	3675–3709 3794–3816	TGAGTCAGCCAGTCTTCGAGAA GCCATCAGTTCCATCACTACCA	141	Primer finder
GLUT4	NM_001359114	429–448 482–501	GATTCCATCCCACAAGGCAC TCATGCCACCCACAGAGAAG	72	(38)
IRS1	NM_010570.4	1117–1131 1159–1177	GAGCGCCCCCAAACG GTCAGCCCGCTTGTTGATG	60	(38)
IRS2	NM_001081212	3214–3233 3285–3302	CAAGAGCCCTGGCGAGTACA CCGCGGATGCCAGTAGTG	88	(38)
TNFα	NM_013693	533–556 435–456	AAGCCTGTAGCCCACGTCGTA GGCACCACTAGTTGGTTGTCTTTG	121	(40)
MCP-1	NM_011333	214–236 404–423	CCCAATGAGTAGGCTGGAGA GCTGAAGACCTTAGGGCAGA	190	(40)
CCR2	NM_009915	36–56 152–174	GCAAGTTCAGCTGCCTGCAA ATGCCGTGGATGAACTGAGGTAA	138	(40)
CD68	NM_001291058	712–736 788–808	CATCAGAGCCCGAGTACAGTCTACC AATTCTGCGCCATGAATGTCC	96	(40)
F4/80	NM_010130	1877–1899 1989–2011	GAGATTGTGGAAGCATCCGAGAC GATGACTGTACCCACATGGCTGA	134	(40)
β-Actin	NM_031144.2	18–37 205–224	CACCCGCGAGTACAACCTTC CCCATACCCACCATCACACC	207	

### Studies on Cultured Predipocytes Derived From the Mice

The process of adipogenesis involves a determination step, in which vascular stromal or stem cells differentiate into preadipocytes, followed by terminal differentiation and maturation into adipocytes (42–44). Since the steps from the preadipocytes can be replicated *in vitro*, we conducted studies on cultured preadipocytes derived from the mice. Preadipocytes were harvested, cultured and induced to differentiate and mature to adipocytes as per the standard procedures (45). Briefly, the epididymal fat pads were removed, minced, and digested using collagenase at 37°C for 40 min. The digest was centrifuged at low speed, and the infranatant just below the floating layer of cells and oil was removed and transferred to a sterile tube. The infranatant was centrifuged at 500 xg for 10 min and the pellet of cells was collected. Red blood cells in the pellet were removed by the use of lysis buffer and the pellet was washed in plating medium and suspended in fresh plating medium (DMEM/F12 containing 10% FBS). Suspended cells were seeded into 24-well sterile culture plates and incubated at 37°C. When cells reached 90% confluence, they were induced to differentiate using differentiation medium (DMEM, FBS 5%, IBMX 0.25 mM, dexamethasone 0.5 mM, insulin 850 nM, indomethacin 100 μM, penicillin and streptomycin). Forty-eight hours later cells were changed to maintenance medium (DMEM, FBS 10%, insulin 17 nM, penicillin and streptomycin) fresh maintenance medium was replaced every 2 days. Altogether, in about a week after treating with induction medium, adipocytes were fully differentiated and matured with accumulation of fat. Cells were fixed in 10% buffered formalin for 1 h, washed with water and stained for 2 h with a working solution of Oil Red O. Cells were then rinsed repeatedly with water and photographed under an inverted microscope. AR-C118925, a potent, selective and competitive antagonist of P2Y_2_ receptor was synthesized in the laboratory of Dr. Christa E. Müller, co-author and supplied to us (46). Stock solutions of AR-C118925 (40 mM in DMSO) were diluted freshly in culture medium before using.

### Studies on 3T3-L1 Cells

The 3T3-L1, a preadipocyte cell line, has become the gold standard for investigating preadipocyte differentiation and maturation into adipocytes. This cell line faithfully recapitulates the steps in differentiation and maturation of preadipocytes into adipocytes. Differentiation is triggered with a standard cocktail of agents to initiate the cascade of synchronous steps in the differentiation process. The 3T3-L1 cell line was purchased from the ATCC (American Type Culture Collection), Manassas, VA, United States. We have established 3T3-L1 cell line cultures in our laboratory, and using a standard protocol successfully induced them to differentiate and maturate into adipocytes, with accumulation of lipids which stained red with Oil Red O. Briefly, 3T3-L1 cells were cultured in DMEM containing 10%FBS. Two days post confluence, cells were cultured in differentiation medium (DMEM containing 10% FBS, 1 μM dexamethasone, 0.5 mM IBMX and 1 μg/ml insulin) for 2 days. After that, cells were cultured in maintenance medium (DMEM containing 10% FBS and 1 μg/ml insulin) until they accumulated fat droplets. Cells were stained with Oil Red O as described for adipocyte cultures above.

### Lipid Tolerance Test

Lipid tolerance test (LTT) was performed using a standard protocol (47). Groups of WT and KO mice (*N* = 6 per group) fed HFD for 14 weeks were fasted overnight (12–14 h), with free access to drinking water. Baseline blood samples (~30 μl) were collected from the saphenous vein from conscious mice into heparinized microtubes (Microvettes®, Sarstedt, Nümbrecht, Germany). Following that, each mouse was injected intraperitoneally with 200 μl of Intralipid 20% (vol/vol) fat emulsion (Sigma Chemical, Co., St. Louis, MO). After that, blood samples (~30 μl each) were collected at 1, 2, and 4-h time points. During this collection period, mice had free access to drinking water, but not food. Collected blood samples were centrifuged and clear plasma was used to assay triglyceride levels.

### Statistical Analysis

All quantitative data were expressed as mean ± SEM. Comparisons among of the group means were made by analysis of variance (ANOVA), followed by the assessment of statistical significance by the Tukey-Kramer Multiple Comparison test. When the means of only two groups were compared, either unpaired *t*-test or Mann-Whitney non-parametric test were used. *P*-values < 0.05 were considered significant. GraphPad Instat (GraphPad Software, La Jolla, CA, United States) was used for statistical analysis.

## Results

### Effect of Feeding High-Fat Diet in WT and P2Y2 KO Mice

**Figure 1:**
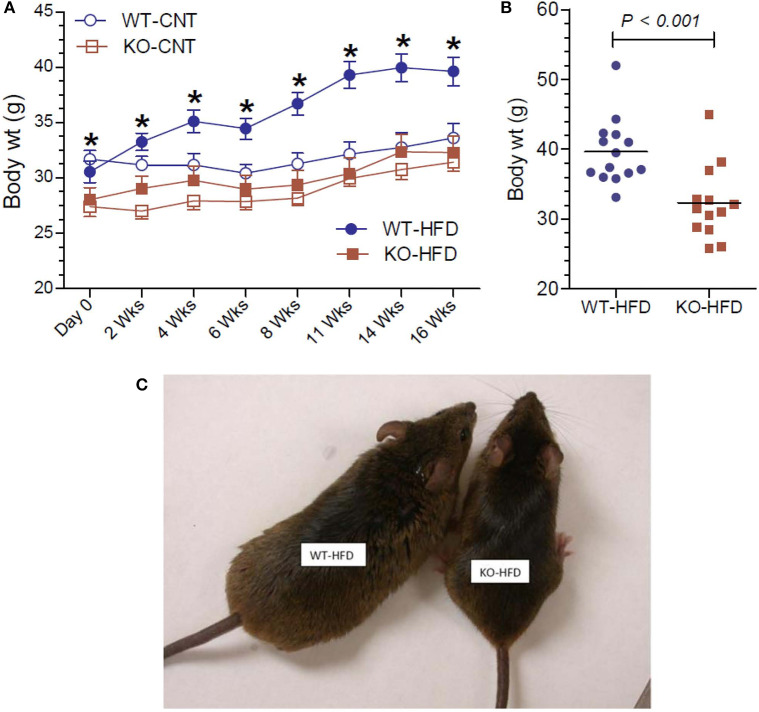
Body weights of WT and P2Y_2_ KO mice fed regular or high-fat diets. **(A)** Shows changes in the mean body weights over the time in WT and P2Y_2_ KO mice fed regular (CNT) or high-fat (HFD) diets. (*N* = 6 mice/genotype in CNT groups and *N* = 14 mice/genotype in HFD groups). *P < 0.05 or better as compared to the corresponding mean weights in the HFD-fed KO mice by direct comparison by unpaired *t*-test. **(B)** Shows scatter graph of terminal body weights in HFD-fed WT and P2Y_2_ KO mice. **(C)** Shows picture of one of the obese WT mice fed HFD vs. an average weighing KO mouse fed HFD after 16 weeks of feeding.

The effect of feeding HFD in WT and KO mice was monitored as a function time for up to 16 weeks and compared with the effect of feeding regular or CNT diet. As shown in Figure 1A, feeding HFD to WT mice resulted in steady and significant increases in the mean body weights as a function of time as compared to CNT-fed WT mice. In fact, significant increases in mean weights in HFD-fed WT mice could be seen as early as 2 weeks. On the contrary, there were no such increases in the mean weights of HFD-fed KO mice as compared to the CNT-fed KO mice. This resulted in a difference of about 22% in the mean weights of HFD-fed WT vs. HFD-fed KO mice at the end of the experimental period. It is interesting to note that even at day 0 the CNT-fed KO mice had significantly lower mean body weight as compared to the age-matched CNT-fed WT mice. This finding suggests a tendency for leanness in P2Y_2_ KO mice. However, based on this finding, one may also argue that since the baseline mean weights in the P2Y_2_ KO mice were significantly lower than the baseline mean weights in the WT mice, the observed increase in the body weights is only relative. However, the mean weight gains in the WT and KO mice fed HFD were 30 and 15%, respectively. The scatter graph (Figure 1B) reveals that despite the expected spread, all the HFD-fed WT mice had terminal body weights higher than the mean body weight in the HFD-fed KO mice. Finally, Figure 1C shows picture of one of the obese WT mice fed HFD next to an average weighing KO mouse fed HFD after 16 weeks of feeding.

### Food Intake and Appearance of Fecal Matter in WT and P2Y2 KO Mice Fed HFD

**Figure 2:**
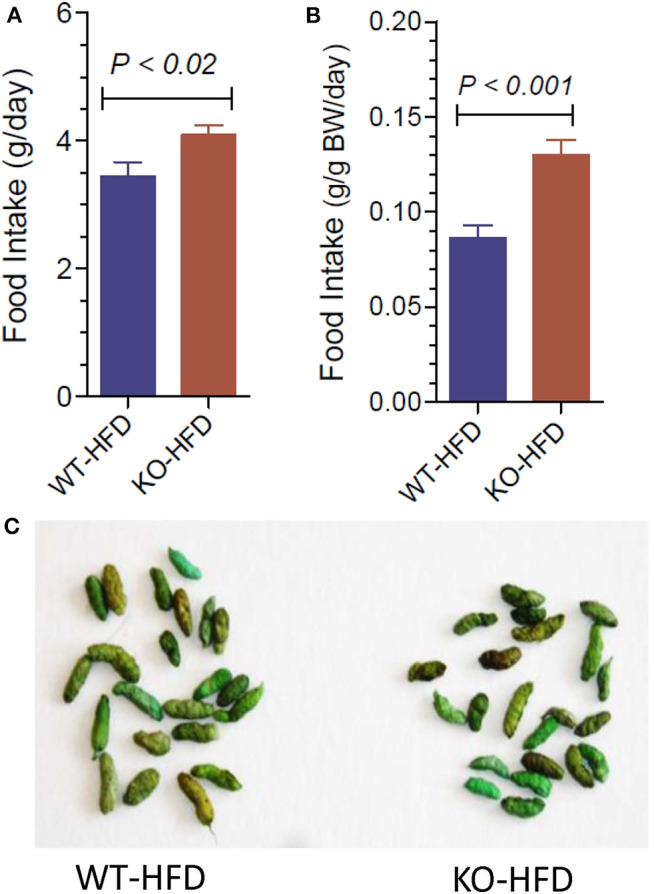
Food intake and appearance of fecal matter in WT and P2Y_2_ KO mice fed HFD. **(A,B)** Show food intake in HFD-fed WT and KO mice at 12 week point expressed as g/day or g/g body weight per day, respectively (*N* = 12 mice per genotype). **(C)** Shows representative samples of well-formed feces in HFD-fed WT and KO mice without any signs of fat or oil. The green color of the feces is due to the dye added to high-fat diet for identification.

Lower body weight could be due to reduced intake of food or loss of undigested fat in the fecal matter, for the observed resistance of the KO mice to HFD-diet feeding. In order to test these possibilities, we determined the 24-h food intake and examined the fecal matter in WT and KO mice (Figure 2A). We found that net food intake was modestly, but significantly higher in the KO vs. WT mice. This difference was pronounced when the data were adjusted to the body weight (Figure 2B). Furthermore, we visually evaluated for signs of loss of undigested or partially digested fat in the feces. For this, fecal matter was collected by placing the mice in metabolic cages. The collected fecal matter was gently placed on an absorbent paper and left for a several minutes at room temperature, and then visually assessed and photographed. This procedure was repeated three times over the course of feeding high-fat diet, in addition to routine observation of fecal matter every 2 weeks in metabolic cages. The criteria of visual evaluation are based on the facts that loss of undigested or partially digested fat results in (i) not well-formed feces, and (ii) stains the absorbent paper with oil. As shown in Figure 2C, none of these two was observed, suggesting that there was no loss of fat in the feces. These data suggest that the observed resistance of the P2Y_2_ KO mice to diet-induced obesity was not due to less consumption of food or loss of undigested food in the feces.

### Morphological Appearance and Terminal Weights of White Adipose Tissue in WT and P2Y2 KO Mice fed HFD

**Figure 3:**
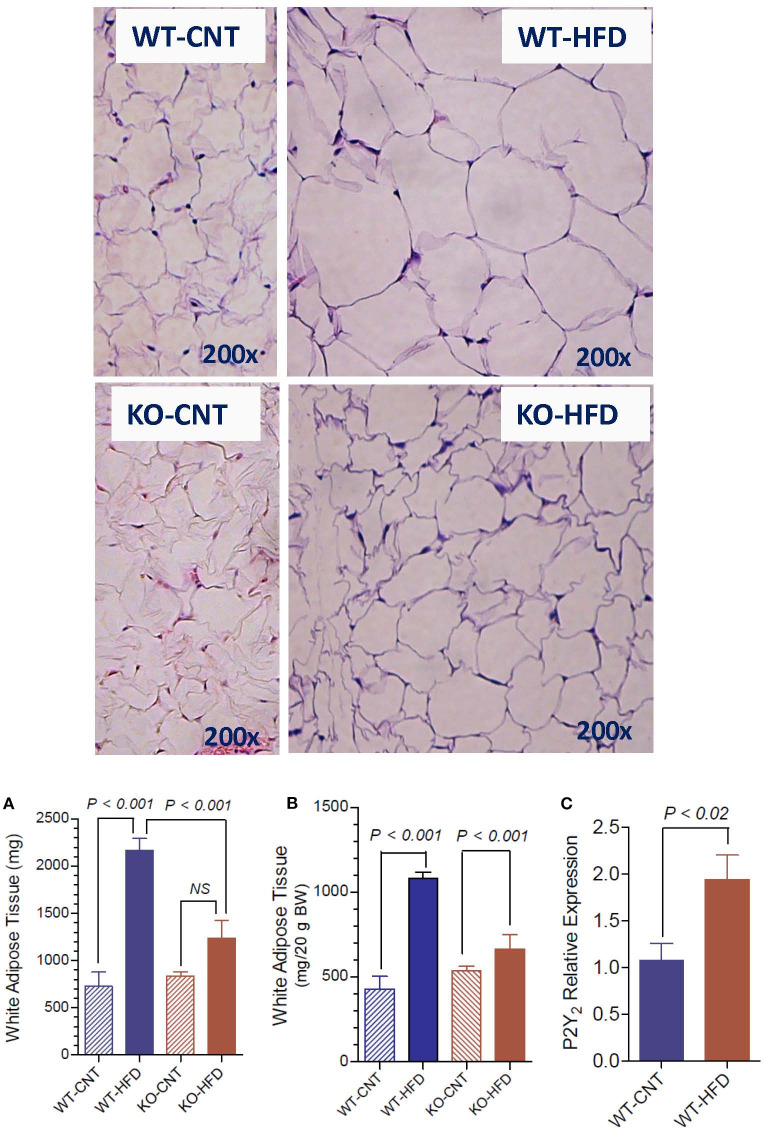
Morphological appearance and mean terminal weights of white adipose tissue in WT and P2Y_2_ KO mice fed regular of high-fat diets. Upper panels show representative profiles of hematoxylin-eosin stained paraffin sections of white adipose tissue from WT and KO mice fed regular (CNT) or high-fat (HFD) diets for 16 weeks. All profiles are shown at the same final magnification (200x). **(A,B)** Show the mean terminal weights of white adipose tissue in WT and KO mice fed regular (CNT) or high-fat (HFD) diets, as mg total weight per mouse or as mg/20 g body weight, respectively (*N* = 6 mice/genotype for CNT; *N* = 12–14 mice/genotype for HFD). Statistics by ANOVA followed by Tukey-Kramer Multiple Comparison Test. **(C)** Shows mRNA expression of P2Y_2_ receptor relative to the mRNA expression of β-actin in the white fat of WT mice fed regular or high-fat diets (*N* = 6 mice/group); statistics by unpaired *t*-test.

Figure 3 upper panels show representative morphology of white adipose tissue in WT and P2Y_2_ KO mice fed regular (CNT) or high-fat (HFD) diets, as determined by hematoxyln-eosin stained sections under light microscope. All panels are displayed at the same final magnification. Feeding HFD to WT mice resulted in large increases in the size of the fat cells. The corresponding increases in KO mice were far less. In fact, the size of the fat cells in the HFD-fed KO mice was similar to the size of the fat cells in the control diet-fed WT mice. Furthermore, in control diet-fed KO mice the fat cells appear to be not fully loaded with fat. Figure 3A,B show terminal mean weights of abdominal white adipose tissue in WT and P2Y_2_ KO mice fed CNT or HFD diets, expressed as total amount per animal or adjusted to body weight, respectively. As shown, feeding HFD to WT mice resulted in significant 2.97- and 2.52-fold increases in the fat content, when expressed as amount per mouse or adjusted to body weight, respectively. On the other hand, the corresponding increases in the KO mice were 1.47- and 1.23-fold, only. Figure 3C shows mRNA expression of P2Y_2_ receptor in white fat increased by ~2-fold in WT mice after feeding HFD. These observations suggest that P2Y_2_ receptor may be playing a role in the adipogenesis and adipose tissue expansion.

### Terminal Serum Insulin, Leptin, and Adiponectin Levels in WT and P2Y2 KO Mice fed HFD

**Figure 4:**
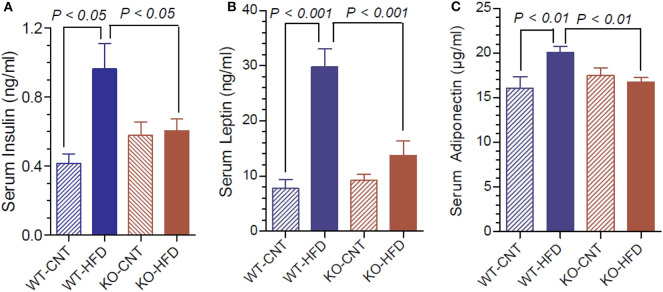
Terminal serum insulin, leptin and adiponectin levels in WT and P2Y_2_ KO mice fed regular or high-fat diets. Serum samples were assayed for insulin, leptin and adiponectin using commercial ELISA or EIA kits. **(A)** Shows serum insulin levels; **(B)** shows serum leptin levels; **(C)** shoes serum adiponectin levels; in WT and P2Y_2_ KO mice fed regular or high-fat diet (*N* = 6 mice/genotype on CNT diet and *N* = 10–13 mice/genotype on HFD diet). Statistics by ANOVA followed by Tukey-Kramer Multiple Comparison Test.

Figure 4A shows that feeding HFD resulted in 2.3-fold higher terminal serum insulin levels in WT, but not in KO mice. Leptin and adiponectin are hormones elaborated by white adipose tissue (adipocytokines). They have several functions in energy homeostasis and regulation. Hence, we determined the terminal serum levels of leptin and adiponectin in the WT and P2Y_2_ KO mice fed CNT or HFD diets. As shown in the Figure 4B, feeding HFD to WT mice resulted in ~4-fold increase in serum leptin levels. The corresponding increase in KO mice was only 1.46-fold. Figure 4C shows that feeding HFD to WT mice resulted in significant 1.25-fold increase in serum adiponectin, whereas there was no change in the serum adiponectin levels in KO mice following HFD feeding.

### Glucose Tolerance Test in WT and P2Y2 KO Mice fed Regular or High-Fat Diets

**Figure 5:**
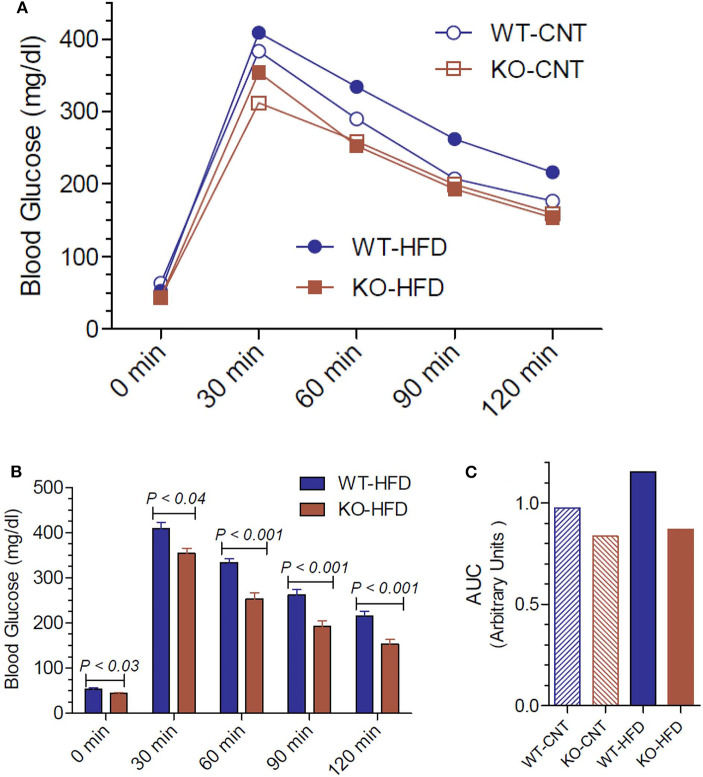
Glucose tolerance test in WT and P2Y_2_ KO mice fed regular or high-fat diet for 14 weeks. Glucose tolerance test was conducted toward the end of the experimental period of feeding high-fat diet. **(A)** Mean blood glucose values in all groups at different time points after administration of glucose. *N* = 5 mice/genotype in CNT diet, *N* = 12 mice/genotype on high-fat diet. **(B)** Blood glucose values in high-fat diet fed WT and KO mice at different time points. *N* = 12 mice/genotype; statistics shown are by direct comparison by unpaired *t*-test. **(C)** Area under curve (AUC) for the data shown in **(A)**.

Since the P2Y_2_ KO mice were resistant to HFD-induced weight gain, we conducted glucose tolerance test (GTT) in WT and P2Y_2_ KO mice fed CNT or HFD diets. Figure 5A shows the GTT curves for all the four groups tested. Figure 5B shows all time points of GTT in HFD-fed WT and KO mice with statistical differences indicated on the bars. As shown in Figure 5B, at all the time points examined, HFD-fed WT mice had significantly higher blood glucose levels as compared to the HFD-fed KO mice. Figure 5C gives the area under curve (AUC) for all the four groups, derived from the data presented in Figure 5A. As shown the HFD-fed WT mice group has the highest value for AUC as compared to all other groups.

### Insulin Tolerance Test and Insulin Receptor Status in WT and P2Y2 KO Mice

**Figure 6:**
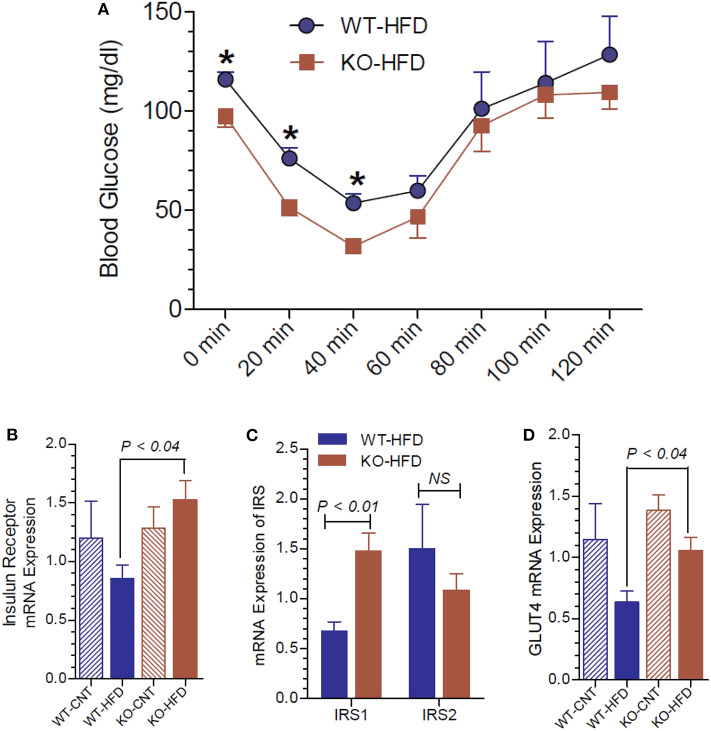
Insulin tolerance test and insulin receptor status in WT and P2Y_2_ KO mice fed regular or high-fat diet for 13 weeks. Insulin tolerance test was conducted toward the end of experimental period of feeding high-fat diet. **(A)** Insulin tolerance test in high-fat diet fed WT and P2Y_2_ KO mice (*N* = 4 mice/genotype). *P < 0.05 compared to the same time point in KO-HFD group. Real-time RT-PCR approach was used to determine the mRNA expression of insulin receptor **(B)**, insulin receptor substrates **(C)**, and GLUT4 **(D)** relative to the expression of β-acting in the white adipose tissues of WT or P2Y_2_ KO mice fed regular (CNT) or high-fat (HFD) diets (*N* = 6 or 7 per group). Statistics by ANOVA followed by Tukey-Kramer Multiple Comparison Test.

To gain further insights into the glucose homeostasis in P2Y_2_ KO mice, we conducted insulin tolerance test (ITT) in WT and P2Y_2_ KO mice fed HFD. As shown in Figure 6A, the KO mice displayed better insulin sensitivity, with significantly lower blood glucose values at two time points following insulin administration. We further probed relative mRNA expression of insulin receptor and insulin receptor substrate (IRS)-1 and−2 in the white adipose tissue of WT and P2Y_2_ KO mice fed regular or HFD. As shown in Figure 6B, although there were no significant differences in the expression of insulin receptor between the WT and KO mice fed regular diet, feeding HFD resulted in a significant difference between these two groups, with KO mice having higher values. In parallel, we observed that the IRS-1 expression levels were significantly higher in the KO mice fed HFD vs. HFD-fed WT mice (Figure 6C). On the other hand, feeding HFD resulted in lower levels of expression of GLUT4 (glucose transporter type-4) in the adipose tissue of both WT and KO mice. However, the decrease was more pronounced in the WT mice, resulting in significantly higher expression levels of GLUT4 in the KO mice vs. WT mice (Figure 6D).

### Expression of Inflammatory Cytokines and Related Molecules in the White Adipose Tissue of HFD-Fed WT and P2Y2 KO Mice

**Figure 7:**
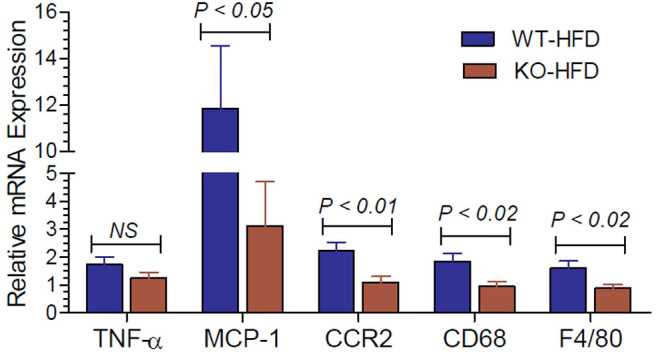
Inflammatory cytokines and related molecules in the white adipose tissue of WT and P2Y_2_ KO mice fed high-fat diet. Real-time RT-PCR approach was used to determine the expression of inflammatory cytokines and related molecules relative to the mRNA expression of β-actin in the white adipose tissue of HFD fed WT and P2Y_2_ KO mice. *N* = 7 mouse samples per group for each parameter. Statistics shown are by direct comparison of the two groups by unpaired *t*-test. TNF-α (tumor necrosis factor-alpha), MCP-1 (monocyte chemoattractant protein-1), CCR2 (C-C chemokine receptor type-2), CD68 (cluster of differentiation 68), and F4/80 (EGF-like module-containing mucin-like hormone receptor-like 1)

Inflammation in the adipose tissue is one of the contributory factors for insulin resistance. Hence, we determined the mRNA expression of inflammatory cytokines and related molecules in the white adipose tissue of HFD-fed WT and P2Y_2_ KO mice. As shown in Figure 7, the expression levels of MCP-1 (monocyte chemoattractant protein-1), CCR2 (C-C chemokine receptor type-2), CD68 (cluster of differentiation 68), and F4/80 (EGF-like module-containing mucin-like hormone receptor-like 1) are significantly higher in the WT mice vs. KO mice after feeding HFD.

### *In vitro* Differentiation and Maturation of Preadipocytes Derived From WT and P2Y2 KO Mice

**Figure 8:**
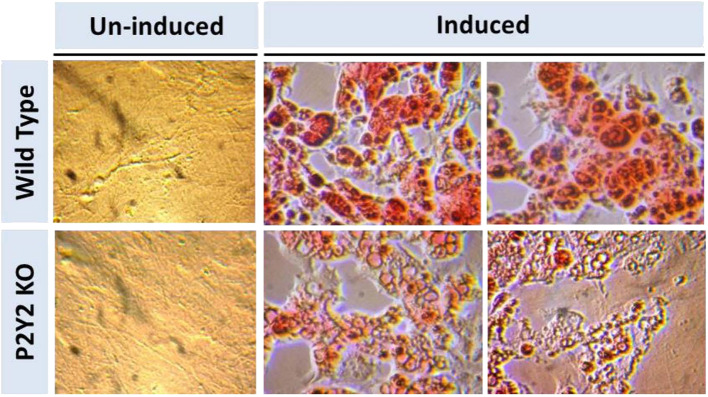
*In vitro* induced differentiation and maturation of pre-adipocytes derived from WT or P2Y_2_ KO mice. Preadipocytes harvested from WT and P2Y_2_ receptor KO mice fed regular diet were induced to differentiate and mature *in vitro*. Matured cells were stained with Oil Red to visualize cellular content of triglycerides. The similar looking dark or gray spots in un-induced panels were apparently due to artifacts in the optical pathway, and were not related to the cultures *per se*, which have distinct appearances.

Figure 8 shows that when preadipocytes from the WT mice were induced to differentiate and mature, there was robust response with accumulation of copious amounts of triglycerides in the mature adipocytes. On the contrary, the differentiation and maturation of preadipocytes derived from the P2Y_2_ KO mice under identical conditions was very weak with scant accumulation of triglycerides in the cells. These observations suggest that P2Y_2_ receptor is needed for the differentiation and maturation of preadipocytes into adipocytes, also known as adipogenesis.

### Suppression of *in vitro* Differentiation and Maturation of Preadipocytes by AR-C118925

**Figure 9:**
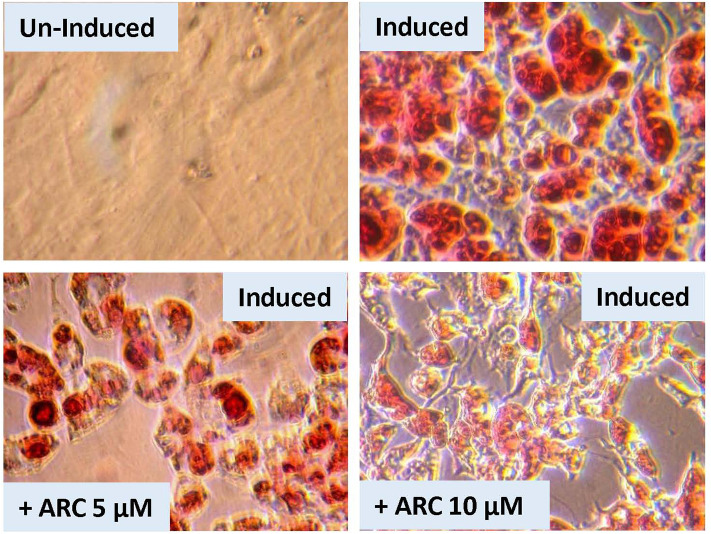
Effect of blockade of P2Y_2_ receptor on *in vitro* differentiation and maturation of pre-adipocytes from WT mice. Preadipocytes harvested from WT mice fed regular diet were induced to differentiate and mature in the absence of presence of AR-C118925, a selective and potent antagonist of P2Y_2_ receptor, at 5 or 10 μM concentration in the culture medium. Cells were stained with Oil Red to visualize cellular content of triglycerides.

There is a possibility that genetic deletion of P2Y_2_ receptor might have resulted in alterations in the expression of other related genes that could impact adipogenesis. In order to test this possibility, we performed an experiment using pharmacological approach to block P2Y_2_ receptor in preadipocytes derived from WT mice. For this, we used AR-C118925, a potent, and selective competitive antagonist of P2Y_2_ receptor. As shown the Figure 9, when preadipocytes derived from WT mice were induced in the presence of 5 or 10 μM of AR-C118925 in the medium, adipogenesis was suppressed to a large extent, with the higher concentration of the drug having a larger effect. At these concentrations, as well as even higher concentrations we tested, AR-C118925 did not cause cell death, and the cells were viable. These observations support a direct role for P2Y_2_ receptor in adipogenesis.

### Suppression of *in vitro* Maturation of 3T3-L1 Cells Into Adipocytes by AR-C118925

**Figure 10:**
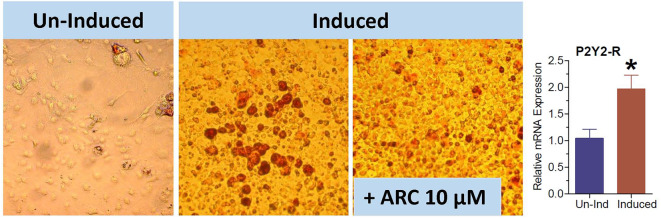
Effect of blockade of P2Y_2_ receptor on differentiation and maturation of cultured 3T3-L1 cells into adipocytes. 3T3-L1 cells were induced to differentiate and mature into adipocytes in the absence or presence of AR-C118925, a selective and potent antagonist of P2Y_2_ receptor at a concentration of 10 μM in the culture medium. Cells were stained with Oil Red to visualize cellular content of triglycerides. Bar graph shows upregulation of P2Y_2_ receptor mRNA during maturation and differentiation of the 3T3-L1 cells into adipocytes (*N* = 4 cultures per condition). *P < 0.02 vs. un-induced group by unpaired *t*-test.

The 3T3-L1 cell line have fibroblast-like morphology, but they differentiate into adipocytes when induced by a cocktail of reagents. We used this cell line to obtain further evidence that the observed effect of genetic deletion or pharmacological blockade of P2Y_2_ receptor on adipogenesis is not cell type-dependent. As shown in Figure 10, the maturation of 3T3-L1 cells into adipocytes with accumulation of triglycerides was markedly suppressed in the presence of 10 μM AR-C118925. Furthermore, as observed in WT mice fed high-fat diet (Figure 3), maturation of 3T3-L1 cells into adipocytes was associated with a 2-fold increase in the mRNA expression of P2Y_2_ receptor (bar graph in Figure 10). These observations lend further supportive evidence that P2Y_2_ receptor has a role in adipogenesis.

### Terminal Serum Triglycerides, Free Fatty Acids, and Glycerol and Lipid Tolerance Test in the WT and P2Y2 KO Mice

**Figure 11:**
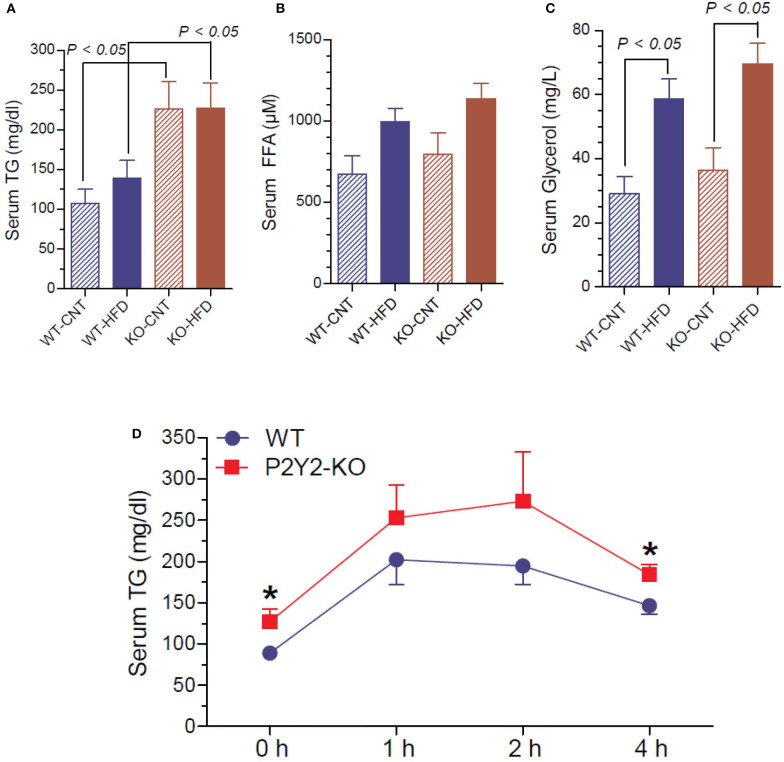
Terminal serum triglycerides, free fatty acids and glycerol and lipid tolerance test in the WT and P2Y_2_ KO mice. Serum samples collected at euthanasia (16-weeks of treatment) were assayed for triglycerides **(A)**, free fatty acids **(B)** and glycerol **(C)** by using commercial assay kits. *N* = 6 samples/genotype for CNT diet, and *N* = 11 or 13/genotype for HFD groups. Statistics by ANOVA followed by Tukey-Kramer Multiple Comparison Test. **(D)** Lipid tolerance test (conducted after 14 weeks of HFD treatment): groups of mice (*N* = 6 mice/genotype) were injected intraperitoneally with 200 μl of Intralipid 20% (vol/vol) fat emulsion, and blood samples were collected at different time points as shown. Plasma samples were analyzed for triglyceride concentration using a commercial colorimetric assay kit. *significantly different from the mean value sin WT mice at the corresponding time point by direct comparison by unpaired *t*-test.

Since P2Y_2_ KO mice were resistant to HFD-induced weight gain, and preadipocytes derived from them show impaired adipogenesis *in vitro*, we determined triglyceride, free fatty acid and glycerol levels in the serum of WT and P2Y_2_ KO mice fed CNT or HFD. As shown in Figure 11A, the serum triglyceride levels were significantly higher in the KO mice fed CNT or HFD as compared to the WT mice fed corresponding diets. No significant differences were seen in the serum free fatty acid levels in different groups of mice, although there is a tendency for increased levels in HFD-fed WT or KO mice (Figure 11B). On the other hand, feeding HFD resulted in significantly higher serum glycerol in both WT and KO mice (Figure 11C). To gain further insights into the lipid metabolism in the WT and KO mice fed with HFD, we conducted intraperitoneal lipid tolerance test (LTT) in these mice. As shown in Figure 11D, we observed slightly better lipid tolerance in WT mice as compared to the KO mice, although the differences between the mean values during the peak were not statistically significant.

### Relative Expression of Lipases and Fatty Acid Translocase in WT and P2Y2 KO Mice Fed Regular or HFD

**Figure 12:**
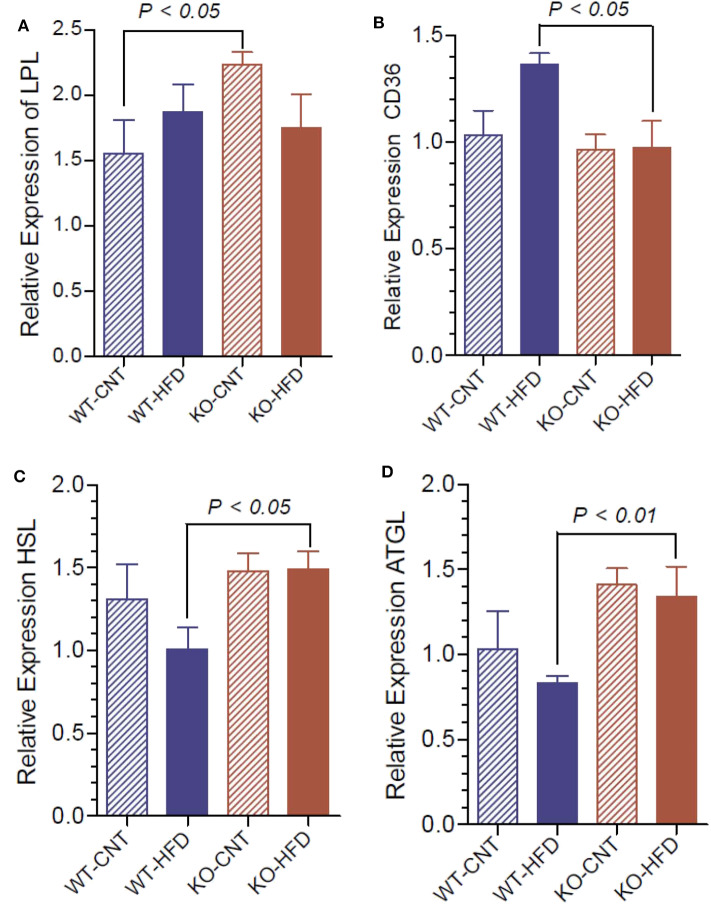
Relative mRNA expression of lipases in the white adipose tissue of WT and P2Y_2_ KO mice fed regular or high-fat diets for 16 weeks. Real-time RT-PCR approach was used to determine the mRNA expression of lipases relative to the mRNA expression of β-actin. **(A)**, lipoprotein lipase (LPL); **(B)**, fatty acid translocase (CD36); **(C)**, hormone-sensitive lipase (HSL); and **(D)**, adipose triglyceride lipase (ATGL). *N* = 6 samples per group. Statistics by ANOVA followed by Tukey-Kramer Multiple Comparison Test.

To understand the lipolytic activity that may explain the observed significant changes in serum triglyceride and glycerol, we determined the mRNA expression of lipoprotein lipase (LPL), hormone-sensitive lipase (HSL), adipose triglyceride lipase (ATGL) and fatty acid translocase (CD36) in white fat of WT and P2Y_2_ KO mice fed CNT or HFD. As shown in Figure 12A, regular diet-fed KO mice had significantly higher levels of LPL as compared to the regular diet-fed WT mice. Such a difference in the expression of LPL in the HFD-fed WT vs. KO mice could not be seen. Feeding HFD to WT mice resulted in significant increases in the CD36 as compared to HFD-fed KO mice (Figure 12B). On the other hand, feeding HFD resulted in significant differences between the WT and KO mice with respect to HSL and ATGL (Figures 12C,D).

## Discussion

In this communication, using a mouse model of genetic deletion of P2Y_2_ receptor, we present compelling experimental evidence that the P2Y_2_ receptor promotes diet-induced obesity. Specifically, we have shown that: (i) P2Y_2_ receptor knockout mice are significantly resistant to HFD-induced obesity or weight gain; (ii) P2Y_2_ receptor promotes HFD-induced inflammation in adipose tissue; (iii) when fed HFD, P2Y_2_ receptor negatively impacts glucose homeostasis; (iv) P2Y_2_ receptor is needed for adipogenesis and expansion of adipose tissue; (v) P2Y_2_ receptor is involved in adipose tissue lipid metabolism; and (vi) the above observed phenomena are not due to reduced consumption of food or loss of undigested fat in the feces (steatorrhea). Hence, we believe that P2Y_2_ receptor is a potential target for the prevention or treatment of diet-induced obesity, the most common form of obesity seen all over the world.

P2Y_2_ receptor is a widely expressed purinergic receptor, and it has been the most studied P2 receptor. RT-PCR performed in our laboratory indicated that P2Y_2_ receptor is expressed in organs or tissues involved in the development of obesity and insulin resistance, such as skeletal muscle, intestines, liver, pancreas, and white and brown adipose tissues (data not shown). The whole-body knockout mice used in this study were generated by targeted mutagenesis of the *P2ry2* gene in mouse embryonic stem cells. No differences were detected between the P2Y_2_ KO and WT mice on histological examination of organs analyzed, including the kidney, heart, testis, pancreas, liver, trachea, lungs, salivary glands and gastrointestinal tract (27, 28). In our experience with these mutant mice for more than a decade, we found that they live a normal and apparently healthy life, and have longevity comparable to the WT mice. We and other investigators have shown that these mice do exhibit renal differences from WT when under various stressors, as previously published (29, 48).

In the current study, we show that KO mice are significantly resistant to high-fat diet (HFD) induced weight gain or obesity. These findings are in agreement with those of Merz et al. (49) who studied the effects of a high-fat/high sugar diet fed for 20 weeks to male P2Y_2_ whole-body KO mice on a different background strain (C57Bl6). Thus, the finding of relatively “lean phenotype” appears robust and independent of background strain, and dietary sugar. Furthermore, we excluded the possibility of reduced food intake or energy loss in the feces. Food consumption was modestly, but significantly higher in the KO mice vs. WT mice, when normalized to body weight, and feces were free from oil. In fact, we noted that for the same age and sex, the KO mice consistently weighed less than the WT mice, which can be observed in this study on day 0 (Figure 1A), i.e., before starting the HFD. In line with the above *in vivo* observations with respect to body weight, the WT mice on HFD showed large and significant increases in the amount of white fat, whereas the corresponding increases in the KO mice was not significant. Morphological examination of white adipose tissue from the WT and KO mice fed regular or high-fat diet also revealed alterations that are consistent with the increased amount of white fat in the WT, but not in the KO mice. The absence of P2Y_2_ receptor in CNT diet-fed KO mice resulted in smaller and poorly filled adipocytes as compared to the CNT diet-fed WT mice. These differences were more pronounced between the HFD-fed WT vs. KO mice. Thus, the morphological examination suggests that P2Y_2_ receptor is needed for the accumulation of lipids and expansion of adipocyte size. Interestingly, the increased amount of white adipose tissue in WT mice was associated with a 2-fold increase in the mRNA expression of P2Y_2_ receptor. This suggest that P2Y_2_ receptor is potentially involved in the expansion of white adipose tissue in response to high-fat diet.

Assay of terminal serum samples showed significantly increased levels of insulin, leptin and adiponectin in HFD-fed WT mice. In contrast, in the HFD-fed KO mice there were no such significant increases. Adiponectin levels may be elevated in the WT-HFD mice due to the development of peripheral adiponectin resistance (50). In general, higher adiponectin levels are associated with improved prognosis in regards to the development of type 2 diabetes mellitus, inflammation, and obesity. Nonetheless, studies conducted in mice specifically, have shown that plasma adiponectin levels can rise over the course of HFD feeding (51), which is consistent with our observations. Thus, WT mice developed many of the standard features of metabolic syndrome after chronic consumption of the high-fat diet, while KO mice were relatively protected. It is unclear from our current study, which, if any of these effects, were direct results of reduced P2Y_2_ signaling in metabolic tissues, e.g., adipose or liver, or rather than general effects of reduced obesity and inflammatory sequelae. Additional studies with cell-specific P2Y_2_ deletion are needed to clarify these mechanisms.

Another interesting aspect of our study is how P2Y_2_ receptor affects glucose homeostasis and insulin sensitivity. HFD-fed WT mice had significantly higher blood glucose levels as compared to HFD-fed KO mice at all the time points of the glucose tolerance test. One can argue that this is because the KO mice were not as obese as the WT mice after feeding HFD. However, the significantly lower mean fasting blood glucose levels in KO mice vs. WT mice suggest that the KO mice are inherently having better glucose homeostasis. The significant differences observed between the WT and KO mice with respect to the insulin tolerance test also suggest that P2Y_2_ receptor may likely to play a significant role in promoting insulin resistance. This is further supported by the higher level of expression of insulin receptor, insulin receptor substrate-1 (IRS-1) and glucose transporter type-4 (GLUT4) in the white adipose tissue of HFD-fed KO mice vs. HFD-fed WT mice.

Both lipid metabolism and inflammation in white adipose tissue are known to be altered in obesity, and contribute for the development of insulin resistance in obese subjects. Hence, we examined the mRNA expression of inflammatory cytokines and associated molecules in the white adipose tissue of WT and KO mice fed HFD. We observed significantly higher expression of MCP-1, CCR2, CD68, and F4/80 in the white fat of WT mice, as compared to the white fat of KO mice. This finding may also contribute to better glucose homeostasis in the KO vs. WT mice fed HFD. In this context, it has been reported that P2Y_2_ receptors induce increased MCP-1 synthesis and release from rat alveolar and peritoneal macrophages (52).

Parallel *in vitro* experiments with preadipocytes derived from the WT or KO mice confirmed that P2Y_2_ receptor is involved in the differentiation and maturation of preadipocytes into adipocytes with accumulation of lipids in the cells. The suppression of *in vitro* differentiation and maturation of preadipocytes derived from WT mice by the competitive P2Y_2_ receptor antagonist, AR-C118925 further validated the involvement of P2Y_2_ receptor in adipogenesis. Finally, the replication of the effect of AR-C118925 in the 3T3-L1 cell line induced to differentiate into adipocytes provides clinching evidence that the effect seen is independent of cell type, but is related to the activity of P2Y_2_ receptor. It is interesting to note that just like adipose tissue *in vivo*, differentiation and maturation of the 3T3-L1 cell line into adipocytes was associated with a 2-fold increase in the P2Y_2_ receptor mRNA expression.

Adipocytes are derived from pluripotent MSC (mesenchymal stromal cells) that have the capacity to develop into several cell types, such as adipocytes, myocytes, chondrocytes, and osteocytes [reviewed in (53)]. These stem cells reside in the vascular stroma of adipose tissue. When appropriately stimulated (e.g., ATP excess states), these stem cells undergo a process of commitment in which the progenitor cells become restricted to adipocyte lineage. Recruitment to this lineage gives rise to preadipocytes, which when induced, undergo mitotic clonal expansion and differentiate into adipocytes. It has been reported that human adipose tissue derived MSC express several purinergic receptors (P2X and P2Y series), but with a predominant role for P2Y_2_ and P2Y_6_ receptors in eliciting intracellular calcium response (54). There is also evidence that purinergic receptors play a role in stem cell differentiation (55). Li et al. (56) concluded that UTP suppresses osteogenic differentiation of bone marrow-derived stromal cells, while enhancing adipogenic differentiation by activating P2Y_2_ receptor. The effect was apparently mediated through the ERK1/2 signaling pathway. Hence, one may conclude that resistance of the P2Y_2_ KO mice to HFD-induced obesity may be due to non-committal of the vascular stromal cells into preadipocytes. Our *in vitro* data where preadipocytes isolated from the P2Y_2_ KO mice showed impaired differentiation and maturation into adipocytes suggests a role for P2Y_2_ receptor in this process. However, one can still argue that there may be fewer preadipocytes in the preparation from the P2Y_2_ KO mice due to non-committal of vascular stromal cells into the preadipocyte lineage. Our *in vitro* data on preadipocytes derived from the WT mice, where AR-C118925 suppressed adipogenesis counters that argument. This is further strengthened by the suppression of differentiation and maturation of 3T3-L1 cells into adipocytes by AR-C118925. Despite the above, it is possible that P2Y_2_ receptor may be playing a role in the committal of vascular stromal cells into adipocyte lineage, in addition to its role in the differentiation and maturation of preadipocytes. This aspect needs further investigation using appropriate methods, which are beyond the scope of this communication. The commitment of MSC toward adipogenic or osteogenic lineage involves various signaling pathways. These pathways include: the β-catenin-dependent Wnt, Hedgehog (Hh) and Bone Morphogenetic Protein (BMP) signaling pathways [reviewed in (57)].

The major energy reserve, the triglycerides (TAG) stored in the white adipose tissue, constantly go through cycles of lipolysis and re-esterification in an orderly manner. This is a complex process involving several hormones, receptors, enzymes and other molecules. Since we observed distinct differences in the morphology of white adipocytes in WT and KO mice, suggesting decreased accumulation of TAG in the adipocytes of KO mice, we assayed terminal serum samples for TAG and its metabolites, namely free fatty acids and glycerol, and conducted an intraperitoneal lipid tolerance test. Analysis of serum samples revealed elevated levels of triglycerides in P2Y_2_ KO mice, irrespective of the dietary regimen, which may reflect altered lipid metabolism in the adipose tissue. However, serum glycerol levels were elevated in both WT and KO mice on HFD relative to control diet, with no genotype difference. Despite these alterations in the serum, the observed differences between HFD-fed WT and KO mice with respect to the lipid tolerance test are minor, and not statistically significant. In fact, if taken as percent changes from baseline or 0 h, these numerical differences were obliterated. It is also possible that the KO mice are able to metabolize lipids more efficiently as compared to the WT mice, as suggested by the metabolic phenotypic data (24). Determination of mRNA expression of key lipases in the white adipose tissue provided some insights into the observed differences in blood lipids. The relatively higher activities of lipoprotein lipase (LPL), hormone-sensitive lipase and adipose triglyceride lipase (ATGL) in KO mice may result in increased blood levels of free fatty acids. However, due to differences in the rates of utilization of free fatty acids between the WT and KO mice, the observed differences in the activities of LPL and ATGL in KO mice might not be reflected in corresponding differences in blood levels of free fatty acids. Based on the current observations, it appears that P2Y_2_ receptor promotes accumulation of TAG in adipocytes, and suppression of this receptor activity enhances lipolysis resulting in release of free fatty acids and glycerol into the blood. Thus, it appears that the observed changes in the lipid metabolism in the KO mice are due to decreased adipogenesis in the absence of P2Y_2_ receptor. Obviously, the altered lipid metabolism in the adipocyte of WT and KO mice fed HFD needs further investigation including its effect on hepatic lipid metabolism.

Recently it has been reported that constitutive P2Y_2_ receptor activity suppresses basal lipolysis in human adipocytes. Ali et al. (58) showed that pharmacological antagonism or knockdown of P2Y_2_ receptor increases intracellular cAMP levels and enhances basal lipolysis. They also observed that acute enhancement of basal lipolysis following P2Y_2_ receptor antagonism alters the profile of secreted adipokines leading to long-term adaptive decrease in lipolysis. Based on their findings, Ali et al. (58) concluded that basal lipolysis and adipokine secretion are controlled by autocrine purinergic signaling in human adipocytes. Our above observations on the alterations in plasma lipids and adipokines in P2Y_2_ receptor KO mice fed high-fat diet are consistent with this report.

Adamson et al. (59) investigated whether mice lacking P2Y_2_ receptor on myeloid cells, achieved by transplanting bone marrow from P2Y_2_ receptor knockout mice into normal mice, are protected against acute and chronic inflammation. Based on their data, they concluded that P2Y_2_ receptor on myeloid cells is important in mediating acute inflammation but is dispensable for the development of whole body insulin resistance in diet-induced obese mice. Thus, it appears that deletion of P2Y_2_ receptor in adipocytes and other organs, is needed to prevent insulin resistance in obese mice. This aspect needs to be further evaluated using cell-specific knockout models of P2Y_2_ receptor.

Finally, a recent review of the involvement of purinergic signaling in obesity by Burnstock and Gentile (60) states that currently, no wholly successful pharmacological treatments are available for obesity and related adverse consequences. It also adds that in recent years, hints obtained from several experimental animal models support the notion that purinergic signaling, acting through ATP-gated ion channels (P2X), G protein-coupled receptors (P2Y), and adenosine receptors (P1), is involved in obesity, both at peripheral and central levels. Thus, while this review provides “proof of concept” for the involvement of purinergic signaling in obesity, our data provide much needed evidence by focusing on the role of P2Y_2_ receptor in diet-induced obesity. Furthermore, a recent report by Negri et al. (61) also confirmed that P2Y_2_ receptor is a regulator of the formation of cardiac adipose tissue and its fat-associated lymphoid clusters. Using the same model of P2Y_2_ receptor knockout mouse, but in C57/Bl6 genetic background, specifically the report presented similar findings as shown in this paper with respect to the role of P2Y_2_ receptor in differentiation and maturation of preadipocytes derived from pericardial fat, and promoting inflammation in pericardial adipose tissue. Thus, it is clear that P2Y_2_ receptor plays a role in adipogenesis and thus diet-induced obesity.

## Conclusion

We demonstrated that genetic deletion of P2Y_2_ receptor significantly protects mice against high-fat diet-induced obesity. Based on the experimental evidence presented, this protection is not due to reduced consumption of food or loss of undigested fat through feces. On the other hand, *in vivo* and *in vitro* data suggest that P2Y_2_ receptor plays a role in adipogenesis and expansion of adipose tissue, adipocyte lipid metabolism, and inflammation in white adipose tissue, and thus promotes glucose intolerance and/or insulin resistance. We consider that this communication represents a significant forward step in our understanding of the pathophysiology of obesity as it relates to purinergic signaling.

## Data Availability Statement

The datasets generated for this study are available on request to the corresponding author.

## Ethics Statement

The animal study was reviewed and approved by Institutional Animal Care and Use Committee of the VA Salt Lake City Health Care System.

## Author's Note

Parts of this work has been presented at the Kidney Week 2013 of the American Society of Nephrology, November 2013, Atlanta, GA, Experimental Biology 2015 meeting, April 2015, Boston, MA, and Kidney Week 2016 of the American Society of Nephrology, November 2016, Chicago, IL, and appeared as printed abstracts in the proceedings of those meetings (23, 24).

## Author Contributions

YZ, CE, and BK were involved in conception and design of research and drafted the manuscript. YZ and BK performed the experiments and prepared the figures. CM synthesized and analyzed AR-C118925 and provided expert advice on its use. LL provide expert advice on induction of adipogenesis *in vitro*. YZ, CE, LL, and BK interpreted results of the experiments. YZ, CE, LL, CM, and BK edited and revised the manuscript and approved the final version of the manuscript.

## Conflict of Interest

The authors declare that the research was conducted in the absence of any commercial or financial relationships that could be construed as a potential conflict of interest.

## Abbreviations

WT: Wild Type
KO: Knockout
CNT: Control diet
HFD: High-fat diet
R: Receptor
BW: Body Weight.

## References

[1] De Lorenzo A Gratteri S Gualtieri P Cammarano A Bertucci P Di Renzo L. Why primary obesity is a disease? J Transl Med. (2019) 17:169. 10.1186/s12967-019-1919-y 31118060 PMC6530037

[2] Almond N Kahwati L Kinsinger L Porterfield D. Prevalence of overweight and obesity among U.S. military veterans. Mil Med. (2008) 173:544–9. 10.7205/MILMED.173.6.544 18595417

[3] Breland JY Phibbs CS Hoggati KJ Washington DL Lee J Haskell S. The obesity epidemic in the veterans health administration: prevalence among key populations of women and men veterans. J Gen Intern Med. (2017) 32:11–7. 10.1007/s11606-016-3962-1 28271422 PMC5359156

[4] Reyes-Guzman CM Bray RM Forman-Hoffman VL Williams J. Overweight and obesity trends among active duty military personnel: a 13-year perspective. Am J Prev Med. (2015) 48:145–53. 10.1016/j.amepre.2014.08.033 25442226

[5] Bray GA. Medical consequences of obesity. J Clin Endocrinol Metab. (2004) 89:2583–9. 10.1210/jc.2004-0535 15181027

[6] Pi-Sunyer X. The medical risks of obesity. Postgrad Med. (2009) 121:21–33. 10.3810/pgm.2009.11.2074 19940414 PMC2879283

[7] Basen-Engquist K Chang M. Obesity and cancer risk: recent review and evidence. Curr Oncol Rep. (2011) 13:71–6. 10.1007/s11912-010-0139-7 21080117 PMC3786180

[8] James WPT McPherson. The cost of overweight. Lancet. (2017) 2:e203–4. 10.1016/S2468-2667(17)30068-3 29253480

[9] Kjellberg J Larsen AT Ibsen R Højgaard B. The socioeconomic burden of obesity. Obesity facts. (2017) 10:493–502. 10.1159/000480404 29020681 PMC5741162

[10] Tremmel M Gerdtham UG Nilsson PM Saha S. Economic burden of obesity: a systematic literature review. Int J Environ Res Public Health. (2017) 14:435. 10.3390/ijerph14040435 28422077 PMC5409636

[11] Kyle TK Dhurandhar EJ Allison DB. Regarding obesity as a disease: evolving policies and their implications. Endocrinol Metab Clin North Am. (2016) 45:511–20. 10.1016/j.ecl.2016.04.004 27519127 PMC4988332

[12] Patel DK Stanford FC. Safety and tolerability of new generation anti-obesity medications: a narrative review. Postgrad Med. (2018) 130:173–82. 10.1080/00325481.2018.1435129 29388462 PMC6261426

[13] Srivastaa G Apovian CM. Current pharmacotherapy for obesity. Nat Rev Endocrinol. (2017) 14:12–24. 10.1038/nrendo.2017.122 29027993

[14] Burnstock G. Introductory overview of purinergic signaling. Front Biol. (2011) 3:896–900. 10.2741/e298 21622101

[15] Burnstock G. Pathophysiology and therapeutic potential of purinergic signaling. Pharmacol Rev. (2006) 58:58–86. 10.1124/pr.58.1.5 16507883

[16] Burnstock G Purinergic signaling: therapeutic developments. Front Pharma. (2017) 8:661 10.3389/fphar.2017.00661 PMC5622197 28993732

[17] Kishore BK Carlson NG Ecelbarger CM Kohan DE Müller CE Nelson RD. Targeting renal purinergic signaling for the treatment of lithium-induced nephrogenic diabetes insipidus. Acta Physiol. (2015) 214:176–88. 10.1111/apha.12507 25877068 PMC4430398

[18] Kishore BK Nelson RD Miller RL Carlson NG Kohan DE. P2Y_2_ receptors and water transport in the kidney. Purinerg Signal. (2009) 5:491–9. 10.1007/s11302-009-9151-5 19319665 PMC2776139

[19] Erb L Weisman GA. Coupling of P2Y receptors to G proteins and other signaling pathways. Wiley Interdiscip Rev Membr Transp Signal. (2012) 1:789–803. 10.1002/wmts.62 25774333 PMC4358762

[20] Soltoff SP Avraham H Avraham S Cantley L. Activation of P2Y2 receptors by UTP and ATP stimulates mitogen-activated kinase activity through a pathway that involves related adhesion focal tyrosine kinase and protein kinase C. J Biol Chem. (1998) 273:2653–60. 10.1074/jbc.273.5.2653 9446569

[21] Vallon V Unwin R Inscho EW Leipziger J Kishore BK. Extracellular nucleotides and P2 receptors in renal function. Physiol Rev. (2020) 1:211–69. 10.1152/physrev.00038.2018 31437091 PMC6985785

[22] Zhang Y Ecelbarger CM Kishore BK Genetic deletion of P2Y_2_ receptor confers significant resistance to development of diet-induced obesity and improves glucose tolerance. J Am Soc Nephrol. (2013) 24:195A.

[23] Kishore BK Zhang Y Heiney K Ecelbarger C P2Y_2_ receptor facilitates high-fat diet induced insulin resistance. FASEB J. (2015) 29.

[24] Zhang Y Ecelbarger CM Müller CE Brandes AU Liu T Lesniewski L Role of P2Y_2_ receptor in adipogenesis and metabolism. J Am Soc Nephrol. (2016) 27:198A.

[25] Kishore B Zhang Y Ecelbarger C Inventors; US Department of Veterans Affairs Assignee Methods for Treating Diet-Induced Obesity by Decreasing and Inhibiting P2Y_2_ Purinergic Receptor Expression or Activity. Washington, DC: USPTO Patent # 10,024.846 (2018).

[26] Kishore BK Zhang Y Ecelbarger CM Inventors; US Department of Veterans Affairs Assignee Composition and Methods for the Prevention and Treatment of Diet-Induced Obesity. Washington, DC: USPTO; United States Patent US 10,107,759, B2(2018).

[27] Cressman VL Lazarowski E Homolya L Boucher RC Koller BH Grubb BR. Effect of loss of P2Y_2_ receptor gene expression on nucleotide regulation of murine epithelial Cl^−^ transport. J Biol Chem. (1999) 274:26461–8. 10.1074/jbc.274.37.26461 10473606

[28] Homolya L Watt WC Lazarowski ER Koller BH Boucher RC. Nucleotide-regulated calcium signaling in lung fibroblasts and epithelial cells from normal and P2Y_2_ receptor. (-/-) mice. J Biol Chem. (1999) 274:26454–60. 10.1074/jbc.274.37.26454 10473605

[29] Zhang Y Sands JM Kohan DE Nelson RD Martin CF Carlson NG. Potential role of purinergic signaling in urinary concentration in inner medulla: insights from P2Y_2_ receptor gene knockout mice. Am J Physiol Renal Physiol. (2008) 295:F1715–24. 10.1152/ajprenal.90311.2008 18829742 PMC2604830

[30] Takahashi M Okimuro Y Iguchi G Nishizawa H Yamamoto M Suda K. Chemerin regulates β-cell function in mice. Sci Rep. (2011) 1:123. 10.1038/srep00123 22355640 PMC3216604

[31] Ayala-Pena VB Scolaro LA Santillan GE. ATP and UTP stimulate bone morphogenetic protein-2,−4 and−5 gene expression and mineralization by rat primary osteoblasts involving PI3K/AKT pathway. Exp Cell Res. (2013) 319:2028–36. 10.1016/j.yexcr.2013.05.006 23707969

[32] Fraulob JC Ogg-Diamantino R Fernandes-Santos C Aguila MB Mandarime-de-Lacerda CA. A mouse model of metabolic syndrome: insulin resistance, fatty liver and non-alcoholic fatty pancreas disease. (NAFPD) in C57BL/6 mice fed a high fat diet. J Clin Biochem Nutr. (2010) 46:212–23. 10.3164/jcbn.09-83 20490316 PMC2872226

[33] Zhang et Y Listhrop R Ecelbarger CM Kishore BK Renal sodium transporter/channel expression and sodium excretion in P2Y_2_ receptor knockout mice fed a high-NaCl diet with/without aldosterone infusion. Am J Physiol Renal Physiol. (2011) 300:F657–68. 10.1152/ajprenal.00549.2010 21190950 PMC4068121

[34] Zhang Y Peti-Peterdi J Müller CE Carlson NG Baqi Y Strasburg DL. P2Y_12_ receptor localizes in the renal collecting duct and its blockade augments arginine vasopressin action and alleviates nephrogenic diabetes insipidus. J Am Soc Nephrol. (2015) 26:2978–87. 10.1681/ASN.2014010118 25855780 PMC4657822

[35] Zhang Y Pop IL Carlson NG Kishore BK. Genetic deletion of P2Y_2_ receptor offers significant resistance to development of lithium-induced polyuria accompanied by alterations in PGE2 signaling. Am J Physiol Renal Physiol. (2012) 302:F70–7. 10.1152/ajprenal.00444.2011 21975874

[36] Sukonina V. Lookene A, Olivecrona T, Olivecrona G. Angiopoietin-like protein 4 converts lipoprotein lipase to inactive monomers and modulates lipase activity in adipose tissue. Proc Natl Acad Sci USA. (2006) 14:17450–5. 10.1073/pnas.0604026103 17088546 PMC1859949

[37] Laugerette F Passilly-Degrace P Patris B Niot I Febbraio M Montmayeur JP. CD36 involvement in orosensory detection of dietary lipids, spontaneous fat preference, and digestive secretions. J Clin Invest. (2005) 115:3177–84. 10.1172/JCI25299 16276419 PMC1265871

[38] Lelliott CJ Medina-Gomez G Petrovic N Kis A Feldmann HM Bjursell M. Ablation of PGC-1beta results in defective mitochondrial activity, thermogenesis, hepatic function, and cardiac performance. PLoS Biol. (2006) 4:e369. 10.1371/journal.pbio.0040369 17090215 PMC1634886

[39] Wilson CH Nikolic A Kentish SJ Shalini S Hatzinkotas G Page AJ. Sex-specific alterations in glucose homeostasis and metabolic parameters during ageing of caspase-2-deficient mice. Cell Death Discov. (2016) 2:16009. 10.1038/cddiscovery.2016.9 27551503 PMC4979492

[40] Tabata M Kadomatsu T Fukuhara S Miyata K Ito Y Endo M. Angiopoientin-like protein 2 promotes chronic adipose tissue inflammation and obesity-related systemic insulin resistance. Cell Metab. (2009) 10:178–88. 10.1016/j.cmet.2009.08.003 19723494

[41] Yuan JS Reed A Chen F Stewart CN Jr Statistical analysis of real-time PCR data. BMC Bioinformatics. (2006) 7:85 10.1186/1471-2105-7-85 16504059 PMC1395339

[42] Arimochi H Sasaki Y Kitamura A Yasutomo K. Differentiation of preadipocytes and mature adipocytes requires PSMB8. Sci Rep. (2016) 6:26791. 10.1038/srep26791 27225296 PMC4880908

[43] Gregoire FM Smas CM Sul HS. Understanding adipocyte differentiation. Physiol Rev. (1998) 78:783–809. 10.1152/physrev.1998.78.3.783 9674695

[44] Rosan ED McDougald OA. Adipocyte differentiation from the inside out. Nat Rev Mol Cell Biol. (2006) 7:885–96. 10.1038/nrm2066 17139329

[45] Hausman DB Park HJ Hausman GJ. Isolation and culture of preadipocytes from rodent white adipose tissue. Meth Mol Biol. (2008) 456:201–19. 10.1007/978-1-59745-245-8_15 18516563

[46] Rafehi M Burbiel JC Attah IY Abdelrahman A Müller CE. Synthesis, characterization, and *in vitro* evaluation of the selective P2Y_2_ receptor antagonist AR-C118925. Purinerg Signal. (2017) 13:89–103. 10.1007/s11302-016-9542-3 27766552 PMC5334202

[47] Kotas ME Jurczak MJ Annicelli C Gillum MP Cline GW Schulman GI. Role of capsase-1 in regulation of triglyceride metabolism. Proc Natl Acad Sci USA. (2013) 110:4810–5. 10.1073/pnas.1301996110 23487794 PMC3607017

[48] Rieg T Bundey RA Chen Y Deschenes G Junger W Insel PA. Mice lacking P2Y_2_ receptors have salt-sensitive hypertension and facilitated renal Na+ and water reabsorption. FASEB J. (2007) 21:3717–26. 10.1096/fj.07-8807com 17575258

[49] Merz J Albrecht P von Garien S Ahmed I Dimanski D Wolf D. Purinergic receptor Y_2_. (P2Y_2_)-dependent VCAM-1 expression promotes immune cell infiltration in metabolic syndrome. Basic Res Cardiol. (2018) 113:45. 10.1007/s00395-018-0702-1 30338362

[50] Engin A. Adiponectin-resistance in obesity. Adv Exp Med Biol. (2017) 960:415–41. 10.1007/978-3-319-48382-5_18 28585210

[51] Bullen JVV Jr Bluher S Kelesidis T Mantzoros CS. Regulation of adiponectin and its receptors in response to development of diet-induced obesity in mice. Am J Physiol Endocrinol Metab. (2007) 292:E1079–86. 10.1152/ajpendo.00245.2006 17164441

[52] Stokes L Surprenant A. Purinergic P2y2 receptors induce increased MCP-1/CCL2 synthesis and release from rat alveolar and peritoneal macrophages. J Immunol. (2007) 179:6016–23. 10.4049/jimmunol.179.9.6016 17947675

[53] Tang QQ Lane MD. Adipogenesis: from stem cell to adipocyte. Annu Rev Biochem. (2012) 81:715–36. 10.1146/annurev-biochem-052110-115718 22463691

[54] Ali S Turner J Fountain SJ. P2Y_2_ and P2Y_6_ receptor activation elicits intracellular calcium responses in human adipose tissue-derived mesenchymal stromal cells. Puriner Signal. (2018) 14:371–84. 10.1007/s11302-018-9618-3 30088129 PMC6298923

[55] Kaebisch C Schipper D Babczyk P Tobiasch E. The role of purinergic receptor in stem cell differentiation. Comp Struct Biotechnol J. (2015) 13:75–84. 10.1016/j.csbj.2014.11.003 26900431 PMC4720018

[56] Li W Wei S Liu C Song M Wu H Yano Y. Regulation of the osteogenic and adipogenic differentiation of bone marrow-derived stromal cells by extracellular uridine triphosphate: the role of P2Y_2_ receptor and ERK1/2 signaling. Int Jo Mol Med. (2016) 37:63–73. 10.3892/ijmm.2015.2400 26531757 PMC4687443

[57] Moseti D Regassa A Kim W-K. Molecular regulation of adipogenesis and potential anti-adipogenic bioactive molecules. Int J Mol Sci. (2016) 17:124. 10.3390/ijms17010124 26797605 PMC4730365

[58] Ali S Turner JJO Fountain SJ. Constitutive P2Y_2_ receptor activity regulates basal lipolysis in human adipocytes. J Cell Sci. (2018) 131:jcs221994. 10.1242/jcs.221994 30333139

[59] Adamson SE Montgomery G Seaman SA Peirce-Cottier SM Leitinger N. Myeloid P2Y2 receptor promotes acute inflammation but is dispensable for chronic high-fat diet-induced metabolic dysfunction. Purinergic Signal. (2018) 14:19–26. 10.1007/s11302-017-9589-9 29086245 PMC5842150

[60] Burnstock G Gentile D. The involvement of purinergic signaling in obesity. Purinerg Signal. (2018) 14:97–108. 10.1007/s11302-018-9605-8 29619754 PMC5940632

[61] Negri I Villamil ED Roeck LD Communi D Horckmans M. P2Y_2_ nucleotide receptor is a regulator of the formation of cardiac adipose tissue and its fat-associated lymphoid clusters. Stem Cells Dev. (2020) 29:100–9. 10.1089/scd.2019.0200 31829837

